# Body Composition Findings by Computed Tomography in SARS-CoV-2 Patients: Increased Risk of Muscle Wasting in Obesity

**DOI:** 10.3390/ijms21134670

**Published:** 2020-06-30

**Authors:** Paola Gualtieri, Carmela Falcone, Lorenzo Romano, Sebastiano Macheda, Pierpaolo Correale, Pietro Arciello, Nicola Polimeni, Antonino De Lorenzo

**Affiliations:** 1 Section of Clinical Nutrition and Nutrigenomics, Department of Biomedicine and Prevention, University of Rome Tor Vergata, 0133 Rome, Italy; paola.gualtieri@uniroma2.it (P.G.); delorenzo@uniroma2.it (A.D.L.); 2 Unit of Radiology, Grande Ospedale Metropolitano Bianchi Melacrino Morelli, 89124 Reggio Calabria, Italy; milafalcone@gmail.com (C.F.); pietroarciello@tin.it (P.A.); 3 School of Specialization in Food Science, University of Rome Tor Vergata, 00133 Rome, Italy; 4 Unit of Intensive Care Medicine and Anesthesia, Grande Ospedale Metropolitano Bianchi Melacrino Morelli, 89124 Reggio Calabria, Italy; nucciomacheda58@gmail.com (S.M.); nicopolimeni@hotmail.it (N.P.); 5 Medical Oncology Unit, Grande Ospedale Metropolitano Bianchi Melacrino Morelli, 89124 Reggio Calabria, Italy; correalep@yahoo.it

**Keywords:** diagnostic imaging, CT scan, body composition, fat, lean, liver attenuation, inflammation, muscle wasting, obesity

## Abstract

Obesity is a characteristic of COVID-19 patients and the risk of malnutrition can be underestimated due to excess of fat: a paradoxical danger. Long ICU hospitalization exposes patients to a high risk of wasting and loss of lean body mass. The complex management precludes the detection of anthropometric parameters for the definition and monitoring of the nutritional status. The use of imaging diagnostics for body composition could help to recognize and treat patients at increased risk of wasting with targeted pathways. COVID-19 patients admitted to the ICU underwent computed tomography within 24 h and about 20 days later, to evaluate the parameters of the body and liver composition. The main results were the loss of the lean mass index and a greater increase in liver attenuation in obese subjects. These could be co-caused by COVID-19, prolonged bed rest, the complex medical nutritional therapy, and the starting condition of low-grade inflammation of the obese. The assessment of nutritional status, with body composition applied to imaging diagnostics and metabolic profiles in COVID-19, will assist in prescribing appropriate medical nutritional therapy. This will reduce recovery times and complications caused by frailty.

## 1. Introduction

The SARS-CoV-2 virus has inflicted a deep wound on the world’s socio-healthcare fabric [1]. In fact, the emergency resulted in complex hospitalizations, in which patients experienced solitude and isolation combined with a difficult management of nutrition and motor activity [2]. The COVID-19 ICU (Intensive Care Unit) hospitalization is prolonged up to about 3 weeks, longer than the ICU ordinary stay. Moreover, recovery times, after awakening and bed-rest, are very long, leading to several short and long term complications [3]. Simultaneously, malnutrition is an underestimated and poorly diagnosed complication and risk factor [4]. A characteristic of COVID-19 patients is obesity [5] and this condition can lead to a dangerous paradox [6]. In fact, the excess fat mystifies the perception of the healthcare professionals about nutritional status and makes the common diagnostic methods unsuitable, such as the BMI. This index misclassifies the patient phenotypes because it does not take body composition into account [7]. In fact, the weight hides the fat mass and water imbalances inside, without adequately representing the muscle mass. In COVID-19 hospitalized patients, the detection of these parameters is limited by necessary logistical requirements [6,8].

Wasting is a common phenomenon in ICUs, but given the long hospital stay, the loss of muscle mass could be deeper if not recognized, monitored, and adequately treated. [9]. COVID-19 must be considered a systemic pathology [10], with repercussions on the whole organism for the deregulated activation of inflammatory pathways, including the cytokine storm [11]. The use of methods for assessing nutritional status, such as the thickness of the subcutaneous fat and the area of some muscles [12,13] in routine instrumental exams in the COVID-19 assessment, such as computed tomography (CT), will help prevent malnutrition and related complications, as the outbreaks could continue until at least 2022 [14]. The primary endpoint was to evaluate the differences in body composition during a period ICU hospitalization in overall, lean, and obese groups. The secondary endpoints were to assess the difference in Liver CT status during a period ICU hospitalization in overall, lean, and obese groups.

## 2. Results

**Figure 1:**
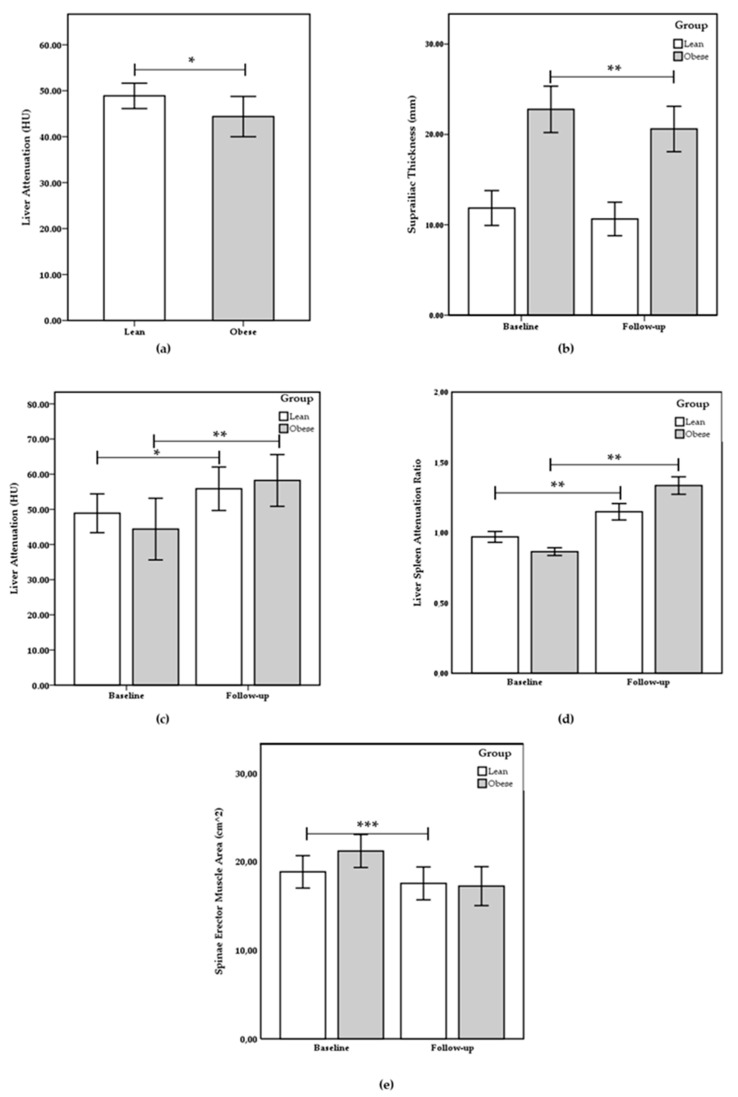
(**a**) Liver attenuation between lean and obese groups at baseline; between baseline and follow up, comparisons for (**b**) suprailiac thickness, (**c**) liver attenuation, (**d**) liver/spleen attenuation ratio, and (**e**) spinae erector muscle cross sectional area are shown. Values are presented as mean ± standard deviation. The statistical significance attributed to results with * *p* < 0.05, ** *p* < 0.005, and *** *p* < 0.001.

Of the 32 patients enrolled in the prospective analytical observation study, 2 subjects were excluded from the study as follows: 2 subjects were excluded due to incomplete data and the absence of the second CT. Finally, 30 patients were included in the study. The age of subjects was 55.40 ± 12.54 years, 36.67% females and 63.33% males. The patients were divided into two groups according to FM (Fat Mass) % and age: 13 lean group (LG) and 17 obese group (OG). At baseline, as reported in Table 1, the subscapular thickness, suprailiac thickness, sum thickness, body density, FM%, and waist circumference were statically increased in the OG compared to the LG (respectively, *p* = 0.001; *p* = 0.006; *p* = 0.000; *p* = 0.001; *p* = 0.001, and *p* = 0.000). Liver attenuation and liver/spleen ratio were statistically reduced in the OG compared to the LG (respectively, *p* = 0.037 and *p* = 0.042). No statistical differences were present for age, spleen attenuation, ESMcsa (Erector Spinae Muscle cross sectional area), and ESM (Erector Spinae Muscle) attenuation between groups. In Table 2, the differences between baseline and follow-up were reported; the subscapular thickness, suprailiac thickness, sum thickness, body density, FM%, waist circumference, and ESMcsa were statistically reduced in the overall and obese groups (*p* < 0.005). In LG, the subscapular thickness, sum thickness, body density, and FM% were statically lower (*p* < 0.05) and suprailiac thickness, waist circumference, and ESMcsa showed no statistical differences. In all groups, liver attenuation and liver/spleen ratio were significantly increased and spleen and ESM attenuation showed no variations (Figure 1). In Table 3, the differences among groups of percentage changes (∆%) between baseline and follow-up were reported. Suprailiac thickness and ESMcsa showed a greater significant reduction in Δ% in the OG (*p* < 0.05). Liver attenuation and liver/spleen ratio showed a larger significant increase in Δ% in the OG (*p* < 0.05).

## 3. Discussion

The main result observed in our sample is the loss of lean mass in obese subjects, together with a higher reduction in the abdominal fat mass indices. In addition, a greater increase in liver density was observed in obese subjects.

The analyzed population had an average age of 55 years, falling in 31.1% of the total COVID-19 cases by age in Italy [15], and there was no statistical difference between the two groups analyzed.

The percentage of men in ICU was higher than women in our sample, as already demonstrated by the data reported by the Italian National Institute of Health [16].

At baseline, the CT derived parameters, related to the fat compartment and waist circumference, were significantly higher in the obese group than in the lean, as expected [17].

Furthermore, lower liver density was observed in obese patients, attributable to a steatosis condition. Given the biological individuality of each subject to the instrumental tests, the measured data were further confirmed using liver/spleen ratio [18].

In obese COVID-19 patients, the presence of ectopic fat in the liver, more represented, is linked to a higher waist circumference [19]. Moreover, the absence of a statistical difference between the two groups, at the baseline, in parameters relating to muscle mass reflects the data from the literature, since the age distribution was overlapping and less than 60 years [20].

The study aimed to observe changes in body compartments in COVID-19 patients at a critical moment, that severely tested hospital and territorial health systems. Infected patients suffered loneliness, developed symptoms of anxiety and insomnia and, in extreme cases, suicide also occurred [21]. In addition to mental distress, their condition was further aggravated by the necessary bed rest and forced hospitalization in confined spaces [22].

These conditions, dependent on COVID-19, worsened psycho-physical abilities, especially muscle performance in the affected population and further aggravated mobility and fragility in the elderly [22].

In ICU, the complex hospitalization conditions were even more extreme in the management of COVID-19 patients.

The observed results reflect about 20 days of ICU admission and the main consequence is wasting, represented by fat and lean mass loss. It is clear that this is attributable to conditions similar to the bed-rest model [23], the inflammatory disease burden and the complex nutrition [24].

The wasting was observed in all examined subjects and they suffered a fat mass loss equal to about 9%. This is attributable to an unusually prolonged increase in energy expenditure.

In the initial period of the acute phase, in response to the injury, an endogenous energy production occurs, between 500 and 1400 kcal/day, which is added to the daily energy expenditure [25]. In the late period of the acute phase, resistance to anabolism occurs, with the consumption of energy and protein reserves, with an increase in expenditure, also in relation to fever and inflammatory status [9].

In COVID-19, the time in the acute phase is longer than 7 days [9], going up to 3 weeks and explaining the severe observed wasting and leading to dysphagia, muscle weakness, and other complications [3].

Moreover, a greater lean mass index loss was observed in obese patients. This can be explained by an increased energy expenditure, insulin resistance, and chronic inflammatory state [26,27].

In the acute phase, in addition to the neuroendocrine response to stress, there is a different release of adipokines, such as leptin, resistin and adiponectin [28].

Leptin, known as the satiety hormone, has an effect on the immune system. Therefore, T, B and natural killer cells use Leptin receptors for their survival [29].

In the acute phase, leptin is co-produced together with the main inflammatory interleukins (IL), IL-6, IL-1, and tumor necrosis factor-α (TNF-α), and acts on the sense of hunger and on the immune response, especially in respiratory infections [30].

An adiposity excess causes insensitivity to leptin, deep anorexia, and a dysregulation of the innate and adaptive immune response, with an increase in susceptibility to respiratory infections [31].

The survival priority determines several metabolic consequences, such as insulin resistance and catabolism [32].

The impairment of the post-receptor pathways of insulin in the liver and the downregulation of glucose transporter-4 GLUT-4 in the muscles are mediated by inflammatory interleukins [33].

This results in hepatic hypergluconeogenesis, impaired muscle glycogen synthesis and lipogenesis in adipose tissue. In addition, to support liver glycogenesis and neoglucogenesis, insulin resistance, inflammation, and neuroendrocrin responses induce muscle proteolysis, to be available to alanine and glutamine, while induces lipolysis to glycerol [32].

The muscle protein breakdown is obtained from an unbalanced upregulation of protein degradation pathways, including the ubiquitin-proteasome system (UPS) [34]. In inflammatory states, UPS transcription is enhanced by the degradation of the inhibitor of transcription factor nuclear factor kappa-light-chain-enhancer of activated B cells (NF-kB) in response to TNF-α action [35,36].

Additionally, myostatin, a member of the transforming growth factor-β family, activates protein breakdown pathways [37].

Therefore, muscle protein synthesis signals, such as the insulin-like growth factor (IGF-1)/protein kinase B (AKT)/mammalian target of rapamycin (mTOR) pathway, are downregulated in acute disease [38].

The triple combination of hypoxemia/hypercapnia, insulin resistance, and oxidative stress, derived from the acute condition, causes severe mitochondrial dysfunction called bioenergetic failure [39].

**Figure 2:**
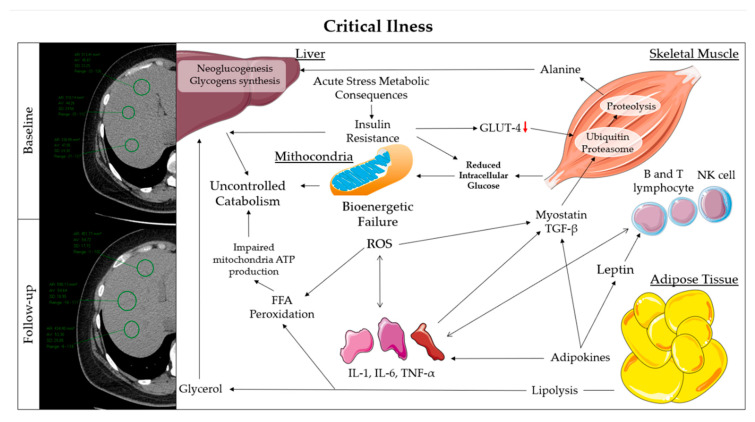
(**Right**) representation of metabolic pathways in response to critical condition. (**Left**) difference to CT images of liver attenuation between baseline and follow-up. FFA: Free Fatty Acids; GLUT: Glucose Transporter; IL: Interleukin; NK: Natural Killer; ROS: Reactive Oxygen Species; TGF: Transforming Growth Factor; TNF: Tumor necrosis factor.

The insufficient glucose uptake and inefficient O_2_/CO_2_ exchange in skeletal muscle causes a collapse of mitochondrial ATP production and the maximum release of oxygen free radicals. These determine further proteolysis, inflammation, and peroxidation of free fatty acids, which can no longer be used as an energy source [40] (Figure 2).

The described phenomena are metabolically complex, and further aggravated in obese subjects, in which many of these processes are already altered. This could help explain the greater loss of ESM in obese subjects.

This data, although preliminary and on a small sample, must alert healthcare professionals.

In fact, corpulence is often reassuring them about the individual’s nutritional status, hiding the wasting. The results obtained show that obese subjects, due to metabolic sequelae, appear more susceptible to the loss of lean body mass, leading to less resilience to disease and rehabilitation. Post COVID-19, obese subjects will be at greater risk of developing a sarcopenia condition and of passing from an obese to an obese sarcopenic phenotype [26,27].

The high increase in liver attenuation in the whole examined sample is attributable to liver gluconeogenesis and glycogen synthesis [41,42].

In obese patients there is an increase of about 15 HU and a greater variation compared to lean ones. Normally, the liver glycogen storage increases liver attenuation by approximately 10 HU [43], as observed in lean subjects.

The greater difference of liver attenuation in obese patients can be attributed to the starting hepatic steatosis condition, which results in a low hepatic attenuation value and uncontrolled glucose overproduction, due to a higher insulin resistance [42].

Unfortunately, the critical working conditions did not allow the measurement of weight and height, consequently making the calculation of the Body Mass Index (BMI) impossible. These parameters are used for the evaluation of nutritional status and, in particular, of malnutrition. The diagnosis of malnutrition, obtained by self-reported parameters, such as the BMI, has poor accuracy and a lack of reliability [44].

The limits of this study are the absence of anthropometric parameters, BMI, and the small sample.

Given the estimation of new increases in SARS-CoV-2 infections [14], we believe it would be useful to study the metabolic profile and sequelae in the future. In particular, insulin resistance and muscle atrophy, due the long hospital stay, and the effectiveness of an early administration of a complete supplement of amino acids should be investigated.

The management of the eating habits, the lifestyle, and the psychological state of these patients will be crucial to prevent the accumulated protein, energy, and psychic debt, from triggering overeating with consequent overtaking of the pre-COVID-19 weight and collateral fattening [45].

## 4. Materials and Methods

### 4.1. Study Design

A retrospective analytical observational study on a single court of adult patients, not previously immunosuppressed, affected by COVID19 pneumonia, came to the emergency department of the Great Metropolitan Hospital “Bianchi Melacrino Morelli” Reggio Calabria, Italy between 19 March and 27 April 2020. Eligible patients were over 18 years of age, with a radiological and molecular diagnosis of COVID 19. Patients with a history of neutropenia, acquired immunodeficiency, who had undergone transplants, or who had received previous immunosuppressive therapies were excluded. The study was approved by the ethical committee of the Great Metropolitan Hospital “Bianchi Melacrino Morelli” Reggio Calabria, Italy (20 April 2020) and informed consent was obtained from all patients enrolled.

### 4.2. Data Collection

The clinical data of all eligible patients were collected and reported prospectively. Data on comorbidities and clinical status have been collected. All the chest CTs were performed at the Great Metropolitan Hospital “Bianchi Melacrino Morelli” Reggio Calabria, Italy. Antiviral, antibiotic, and medica nutritional therapy was started in the emergency department and continued in the clinical wards according to current hospital guidelines. The collected data refer to the same patients enrolled and subsequently re-evaluated.

### 4.3. Definition

A chest CT (GE Medical SYSTEMS Optima) without intravenous contrast was performed within 24 h of admission to the emergency department, baseline, and on about the twentieth day (mean ± SD: 20.3 ± 3.4 days) according to clinical needs, follow-up. Patients were classified as lean and obese, according to Percentage of Fat Mass (FM%) and age according to De Lorenzo et al. [46]. The primary endpoint was to evaluate the differences in body composition during a period ICU hospitalization in overall, lean, and obese groups. The secondary endpoints were used to assess the difference in liver CT status during a period ICU hospitalization in overall, lean, and obese groups.

Fat mass percentage content, anthropometric measurements, liver attenuation, and Erector Spinae Muscles cross sectional area.

To determine the FM%, we used the Siri equation [47]. Body density was obtained by the equation of Durnin et al., using two subcutaneous fat thicknesses of the chest, suprascapular and suprailiac, and the correction factors according to age, sex, and folds used [48]. The subcutaneous fat thickness was measured at CT, given the agreement between the CT and Plicometry method [12]. The subscapular fat thickness was measured in a cross section starting from the origin of the scapular spine on the posterior medial edge up to the skin. The suprailiac fat thickness was measured in a 2 cm cross section from the last rib on the middle axillary line up to the skin. The subcutaneous fat thickness parameters measured on CT were doubled before insertion into the equation of Durnin et al. [48]. On CT images, hepatic steatosis was defined according to Wells et al. [18]. Liver attenuation was an average of four measurements in segments 3, 5, 6, and 7. Spleen attenuation was an average of three anterior, central, and posterior measurements. Waist circumference was measured at the last rib. Where part of the abdomen was outside the field of the image, waist circumference was estimated with a continuous arc. All CTs were performed with patients in the supine position with arms folded and hands positioned under the nape of the neck. All the measurements on CT were conducted in duplicate by two different operators. If differences between the measures of more than 5% were detected, a third operator was asked to repeat the operation. At the level of the T12 vertebra, the Erector Spinae Muscles cross sectional area (ESMcsa) was measured according to Tanimura et al. [13].

### 4.4. Statistical Analysis

The inputs for the calculation of the sample size were a difference between two dependent means (matched pairs), a power of 85%, a significance level of 5% (two-tails), and the detection of an effect size of 0.5 between the pairs. According to the study setting, the sample size was 27 and the G * Power software (Version 3.1.9.6, Germany) was used [49]. Since 20% of the sample may not have all the expected parameters, 32 subjects were enrolled.

All statistical analyzes were conducted with SPSS 23 software (version 23.0, IBM, Armonk, NY, USA). The data collected before statistical evaluations were analyzed for the presence of outliners and for normally distribution with the Shapiro–Wilk test, and all variables had a normal distribution. The data presented are expressed as mean, standard deviation, and as the percentage change between baseline and follow up (∆%). Before, the differences between lean and obese subjects were assessed at baseline with the *T*-test for independent samples. Subsequently, in overall, lean, and obese, the differences in the groups were assessed between baseline and follow-up with the *T*-test for matched pairs. Conclusively, the differences in ∆% between baseline and follow up among groups were assessed with the Anova test. Statistical significance was set to a value of *p* < 0.05. All *p* values shown are two-tailed.

## 5. Conclusions

Intensive care management of critically ill patients with COVID-19 requires a rigorous and multidisciplinary approach. Evidence-based medicine should be applied, including personalized nutrition and rehabilitation therapy, in order to minimize muscle wasting functional decline.

## Figures and Tables

**Table 1:** Descriptive and Compared between groups at baseline.

Parameters	Overall (*n* 30)	Lean (*n* 13)	Obese (*n* 17)	
	Mean ± SD	Mean ± SD	Mean ± SD	*p*
Subscapular Thickness (mm)	16.45 ± 6.92	10.85 ± 3.81	21.36 ± 4.95	0.001
Suprailiac Thickness (mm)	17.68 ± 8.32	11.85 ± 5.11	22.77 ± 7.25	0.006
Sum Thickness (mm)	34.13 ± 13.95	22.70 ± 8.35	44.13 ± 9.21	0.000
Body Density (kg/m^3^)	1.03 ± 0.01	1.05 ± 0.01	1.03 ± 0.01	0.001
Fat Mass (%)	28.42 ± 6.29	23.34 ± 5.21	32.87 ± 2.73	0.001
Waist Circumference (cm)	103.19 ± 10.18	93.72 ± 4.10	111.48 ± 4.97	0.000
Liver Attenuation (HU)	46.17 ± 4.91	48.89 ± 2.76	43.79 ± 5.28	0.037
Spleen Attenuation (HU)	49.68 ± 6.85	51.84 ± 6.27	47.78 ± 7.15	0.267
LRS	0.91 ± 0.10	0.97 ± 0.10	0.86 ± 0.08	0.042
ESMcsa (cm^2^)	20.08 ± 4.52	18.84 ± 4.42	21.18 ± 4.63	0.387
ESM attenuation (HU)	27.63 ± 3.24	28.54 ± 5.84	26.83 ± 4.29	0.667

Differences among groups at baseline. All parameters are presented as mean ± standard deviation and were compared by *T*-test for independent samples. Statistical significance was attributed as *p* < 0.05. LSR: Liver Spleen Ratio. ESM: Erector Spinae Muscle.

**Table 2:** Comparison between baseline and follow-up in each group.

Parameters	Overall			Lean			Obese		
	Baseline	Follow-up		Baseline	Follow-up		Baseline	Follow-up	
	Mean ± SD	Mean ± SD	*p*	Mean ± SD	Mean ± SD	*p*	Mean ± SD	Mean ± SD	*p*
Subscapular Thickness (mm)	16.45 ± 6.92	14.97 ± 7.42	0.000	10.85 ± 3.81	9.01 ± 5.17	0.004	21.36 ± 4.95	19.94 ± 4.84	0.001
Suprailiac Thickness (mm)	17.68 ± 8.32	16.08 ± 7.27	0.004	11.85 ± 5.11	10.64 ± 4.14	0.237	22.77 ± 7.25	20.6 ± 6.15	0.004
Sum Thickness (mm)	34.13 ± 13.95	31.05 ± 13.34	0.000	22.7 ± 8.35	19.65 ± 8.52	0.025	44.13 ± 9.21	40.54 ± 7.72	0.001
Body Density (kg/m^3^)	1.03 ± 0.01	1.05 ± 0.02	0.000	1.05 ± 0.01	1.07 ± 0.01	0.000	1.03 ± 0.01	1.04 ± 0.01	0.000
Fat Mass (%)	28.42 ± 6.29	20.15 ± 7.01	0.000	23.34 ± 5.21	14.5 ± 6.45	0.000	32.87 ± 2.73	24.86 ± 2.54	0.000
Waist Circumference (cm)	103.19 ± 10.18	99.85 ± 9.64	0.002	93.72 ± 4.10	92.25 ± 3.78	0.185	111.48 ± 4.97	108.71 ± 5.55	0.000
Liver Attenuation (HU)	46.17 ± 4.91	57.14 ± 3.48	0.000	48.89 ± 2.76	55.85 ± 3.09	0.017	43.79 ± 5.28	58.22 ± 3.68	0.002
Spleen Attenuation (HU)	49.68 ± 6.85	46.31 ± 5.49	0.013	51.84 ± 6.27	49.02 ± 4.60	0.066	47.78 ± 7.15	44.05 ± 5.47	0.147
LRS	0.91 ± 0.10	1.25 ± 0.17	0.000	0.97 ± 0.10	1.15 ± 0.13	0.004	0.86 ± 0.08	1.34 ± 0.15	0.001
ESMcsa (cm^2^)	20.08 ± 4.52	17.36 ± 3.72	0.000	18.84 ± 4.42	17.52 ± 3.46	0.091	21.18 ± 4.63	17.22 ± 5.11	0.000
ESM attenuation (HU)	27.63 ± 3.24	26.80 ± 6.86	0.510	28.54 ± 5.84	27.29 ± 4.11	0.404	26.83 ± 4.29	26.08 ± 4.82	0.950

**Table 3:** Percentage change presentation and difference between groups.

	∆% Baseline Follow-up			
Parameters	Overall (*n* 30)	Lean (*n* 13)	Obese (*n* 17)	
	Mean ± SD	Mean ± SD	Mean ± SD	*p*
Subscapular Thickness (∆%)	−15.18 ± 11.75	−17.55 ± 16.07	−11.74 ± 4.16	0.182
Suprailiac Thickness (∆%)	−12.38 ± 11.64	−9.54 ± 15.98	−14.75 ± 7.21	0.045
Sum Thickness (∆%)	−14.14 ± 6.32	−14.84 ± 8.48	−13.56 ± 4.62	0.752
Body Density (∆%)	1.88 ± 0.21	1.88 ± 0.28	1.87 ± 0.15	0.953
Fat Mass (∆%)	−28.85 ± 4.66	−31.73 ± 5.03	−26.46 ± 2.83	0.055
Waist Circumference (∆%)	−2.25 ± 2.19	−1.52 ± 2.74	−3.11 ± 0.9	0.206
Liver Attenuation (∆%)	27.61 ± 18.58	14.14 ± 8.45	38.84 ± 17.35	0.018
Spleen Attenuation (∆%)	−4.39 ± 4.76	−4.87 ± 4.42	−3.99 ± 5.42	0.777
LRS (∆%)	40.56 ± 24.79	20.00 ± 6.98	57.7 ± 20.38	0.004
ESMcsa (∆%)	−14.99 ± 15.36	−7.41 ± 9.43	−18.63 ± 6.96	0.031
ESM attenuation (∆%)	−3.03 ± 3.42	−4,37 ± 7.51	−2.79 ± 3.56	0.683

Differences among groups of percentage change (∆%) between baseline and follow-up. All parameters are presented as mean ± standard deviation and were compared by Anova Test. Statistical significance was attributed as *p* < 0.05. LSR: Liver Spleen Ratio. ESMcsa: Erector Spinae Muscle cross-section area.

## Abbreviations

CT	Computed Tomography
ICU	Intensive Care Unit
LG	Lean Group
OG	Obese Group
ESM	Erector Spinae Muscle
ESMcsa	Erector Spinae Muscle cross sectional area
IL	Interleukin
TNF-α	Tumor Necrosis Factor-α
GLUT-4	Glucose Transporter-4
UPS	Ubiquitin-Proteasome System
IGF-1	Insulin-like Growth Factor
AKT	Protein Kinase B
mTOR	mammalian Target Of Rapamycin
HU	Hounsfield Unit
BMI	Body Mass Index
